# VBIT-4 Rescues Mitochondrial Dysfunction and Reduces Skeletal Muscle Degeneration in a Severe Model of Duchenne Muscular Dystrophy

**DOI:** 10.3390/ijms26188845

**Published:** 2025-09-11

**Authors:** Mikhail V. Dubinin, Anastasia E. Stepanova, Irina B. Mikheeva, Anastasia D. Igoshkina, Ekaterina N. Kraeva, Alena A. Cherepanova, Eugeny Yu. Talanov, Anna V. Polikarpova, Maxim E. Astashev, Vyacheslav A. Loginov, Tatiana V. Egorova

**Affiliations:** 1 Department of Biochemistry, Cell Biology and Microbiology, Mari State University, pl. Lenina 1, 424001 Yoshkar-Ola, Russia; lady.stepanowa2010@yandex.ru (A.E.S.); anastasi.igoshkina@yandex.ru (A.D.I.); kkraeva49@gmail.com (E.N.K.); alyona_2022@bk.ru (A.A.C.); 2 Laboratory of Experimental Neurobiology, Institute of Theoretical and Experimental Biophysics, Russian Academy of Sciences, 142290 Pushchino, Russia; mikheirina@yandex.ru; 3 Laboratory of Mitochondrial Transport, Institute of Theoretical and Experimental Biophysics, Russian Academy of Sciences, Institutskaya 3, 142290 Pushchino, Russia; evg-talanov@yandex.ru; 4 Laboratory of Modeling and Gene Therapy of Hereditary Diseases, Institute of Gene Biology Russian Academy of Sciences, Vavilova 34/5, 119334 Moscow, Russia; a.polikarpova.marlin@gmail.com (A.V.P.); v.loginov.marlin@gmail.com (V.A.L.); egorovatv@genebiology.ru (T.V.E.); 5 Marlin Biotech LLC, Triumfalny Ave. 1, Sirius, 354340 Sochi, Russia; 6 Federal Research Center “Pushchino Scientific Center for Biological Research of the Russian Academy of Sciences”, Institute of Cell Biophysics, Russian Academy of Sciences, Institutskaya 3, 142290 Pushchino, Russia; astashev@yandex.ru; 7 Prokhorov General Physics Institute, Russian Academy of Sciences, Vavilov Str. 38, 119991 Moscow, Russia

**Keywords:** Duchenne muscular dystrophy, skeletal muscle mitochondria, calcium overload, VDAC, VBIT-4, proteotoxic stress

## Abstract

Duchenne muscular dystrophy (DMD) is a severe X-linked recessive disorder caused by mutations in the *DMD* gene, leading to progressive muscle degeneration and fibrosis. A key pathological feature of DMD is mitochondrial dysfunction driven by calcium overload, which disrupts oxidative phosphorylation and triggers cell death pathways. This study shows the therapeutic potential of VBIT-4, a novel inhibitor of the mitochondrial voltage-dependent anion channel (VDAC), in two dystrophin-deficient mouse models: the mild *mdx* and the severe D2.DMDel8-34 strains. VBIT-4 administration (20 mg/kg) reduced mitochondrial calcium overload, enhanced resistance to permeability transition pore induction, and improved mitochondrial ultrastructure in D2.DMDel8-34 mice, while showing negligible effects in *mdx* mice. VBIT-4 suppressed mitochondrial and total calpain activity and reduced endoplasmic reticulum stress markers, suggesting a role in mitigating proteotoxic stress. However, it did not restore oxidative phosphorylation or reduce oxidative stress. Functional assays revealed limited improvements in muscle strength and fibrosis reduction, exclusively in the severe model. These findings underscore VDAC as a promising target for severe DMD and highlight the critical role of mitochondrial calcium homeostasis in DMD progression.

## 1. Introduction

Duchenne muscular dystrophy (DMD) is the most common X-linked recessive disorder caused by mutations in the *DMD* gene, which encodes the protein dystrophin. Primarily, the disease affects males, occurring in 1 in 3500–5000 newborns, and is marked by progressive muscle weakness resulting in disability and fatal outcomes from respiratory and cardiac failure. No effective genetic therapy for a complete cure for this disorder currently exists [1].

Dystrophin plays a critical role by linking the cytoskeleton of muscle fibers (or cardiomyocytes) to the sarcolemma. In addition, it interacts with ion channels, signaling molecules, scaffold proteins, and the extracellular matrix through the dystrophin-associated protein complex (DAPC). This complex is vital for maintaining the structural integrity of muscle tissue and ensuring its normal physiological function [1,2].

Dystrophin deficiency disrupts the spatial localization of numerous proteins and signaling molecules and induces microdamage to the membrane during contractions, triggering pathological Ca^2+^ infiltration into the cytosol. This process is further exacerbated by the impaired function of sarcolemmal calcium transport channels. The resulting sustained hypercalcemia, observed in both patient muscles and animal models, activates calpains—calcium-dependent proteases that initiate intracellular protein degradation. In addition, calcium-dependent phospholipases are thought to become activated, leading to impaired membrane organization. Collectively, these processes cause the proteolysis of structural components, proteotoxic and oxidative stress, muscle fiber necrosis, secondary inflammation, and fibrosis [1,2].

Sarcolemmal calcium channel blockers can partially reduce, but not completely prevent, calcium overload in dystrophin-deficient muscle fibers, suggesting additional dysfunction in the intracellular calcium handling machinery [3]. Excessive calcium overload in dystrophin-deficient muscles is also caused by the impaired function of sarcoplasmic reticulum (SR) ion channels, which facilitates calcium release from this main Ca^2+^ storage compartment [4]. Under these conditions, excess Ca^2+^ accumulates in the mitochondria, which normally play only a secondary role in calcium buffering [5]. Quantitative measurements confirm that skeletal muscle mitochondria become significantly overloaded with calcium ions under conditions of dystrophin deficiency or other DAPC component disruptions [6,7,8,9,10,11]. This leads to impaired mitochondrial electron transport chain function and mitochondrial permeability transition (MPT) pore opening, triggering various forms of cell death [12]. Studies using experimental models have shown that preventing calcium-induced damage to mitochondria, either by inhibiting the transport of calcium from the SR to mitochondria [12,13,14,15] or by blocking the MPT pore [16,17,18,19,20,21], results in improved mitochondrial function, enhanced skeletal muscle characteristics, and better quality of life in model organisms.

One of the key regulators of mitochondrial function is the voltage-dependent anion channel (VDAC) in the outer mitochondrial membrane, which participates in forming membrane contact sites with the SR [22]. This protein mediates the transport of adenine nucleotides, metabolites, and Ca^2+^ across the mitochondrial membrane while also playing a crucial role in apoptosis [23]. Emerging evidence positions VDAC as a potential therapeutic target for normalizing mitochondrial function in DMD [24]. Recent studies have demonstrated that its inhibition by olesoxime partially improves mitochondrial function and skeletal muscle condition in dystrophin-deficient mice [25].

In this study, we evaluated the in vivo effects of VBIT-4, a novel VDAC inhibitor, on skeletal muscle mitochondrial dysfunction and disease progression in dystrophin-deficient mice. This small-molecule inhibitor has previously demonstrated VDAC-mediated therapeutic effects in Alzheimer’s disease [26,27], lupus [28], diabetes [29], and cardiac fibrosis [30]. We used two different dystrophin-deficient models—classic *mdx* mice with moderate symptoms and D2.DMDel8-34 mice (D2.DMD) with severe progression similar to DMD—and found that intraperitoneal administration of VBIT-4 (20 mg/kg) delayed the development of pathology in D2.DMDel8-34 mice but had no effect on the *mdx* phenotype with mild symptoms. These results confirm the critical role of mitochondrial dysfunction in DMD progression and highlight the therapeutic potential of VDAC-targeted strategies.

## 2. Results

Mouse models of Duchenne muscular dystrophy have a mild disease phenotype. In contrast to patients with DMD and larger animal models, dystrophin-deficient mice do not experience a reduced lifespan and remain mobile until death. To approximate the course of the disease in mouse models to that in patients, additional mutations are introduced. The genetic background can significantly affect the progression of DMD. It has previously been shown that the use of the DBA/2 line as a genetic background significantly aggravates the phenotype of mdx mice [31,32]. In this work, we transferred the previously obtained deletion of exons 8–34 in the *DMD* gene to the DBA/2 background [33] and used D2.DMDel8-34 mice as an example of a severe DMD phenotype. In parallel, the generally accepted mdx model, which has a mild phenotype, was used.

### 2.1. VBIT-4 Reduces Calcium Overload and Enhances Resistance to MPT Pore Induction in the Quadriceps Mitochondria of D2.DMDel8-34 Mice but Not in Mdx Mice

**Figure 1:**
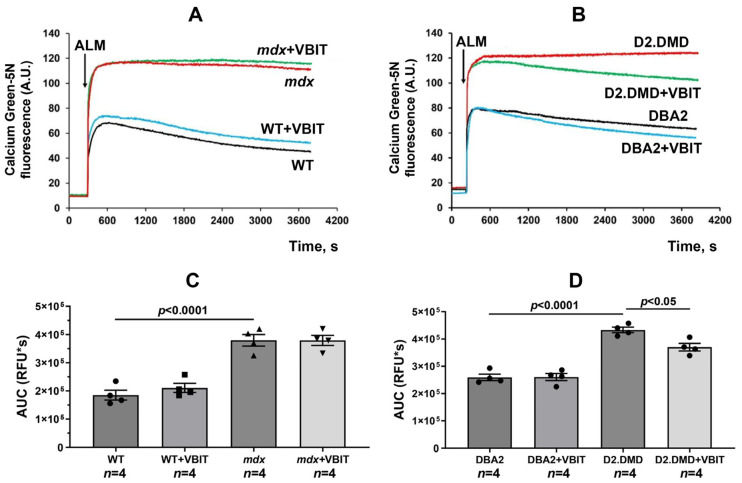
Effect of VBIT-4 on calcium levels in skeletal muscle mitochondria of mice. (**A**,**B**) Representative traces of calcium green-5N fluorescence intensity in mitochondria isolated from quadriceps muscles of *mdx* and WT mice (**A**) and D2.DMD and DBA/2 mice (**B**). Alamethicin (ALM, 0.1 mg/mL) was added (as indicated by the arrow) to induce maximal calcium release from the mitochondrial matrix. The data presented are from a representative experiment conducted on one mitochondrial sample. Similar results were obtained in three additional independent experiments. (**C**,**D**) Quantification of the total calcium release, presented as the AUC of the fluorescence traces. Data are expressed as mean ± SEM.

Modulation of mitochondrial VDACs significantly affects organelle functional activity. We evaluated the effects of VBIT-4 on the functional parameters of skeletal muscle mitochondria in an in vivo experiment. Calcium overload in dystrophin-deficient muscle fibers is accompanied by the excessive accumulation of this ion in the mitochondrial matrix, promoting organelle dysfunction [11,15]. Figure 1 shows that the addition of the channel-forming agent alamethicin to skeletal muscle mitochondria from *mdx* mice resulted in a substantial increase in the calcium green-5N fluorescence intensity compared with the wild-type (WT) controls, indicating significant Ca^2+^ release from the organelles (Figure 1A). Quantification of the total calcium release (area under the curve, AUC) confirmed a significant increase in *mdx* mitochondria compared to WT (Figure 1C). A similar pattern was observed in D2.DMD mice displaying a severe pathology phenotype, where the calcium green-5N fluorescence intensity and AUC were also higher in permeabilized skeletal muscle mitochondria compared with that in DBA/2 control mice (Figure 1B,D). VBIT-4 treatment had no effect on either the fluorescence kinetics or the AUC in *mdx* mice (Figure 1A,C). However, mitochondria from VBIT-4-treated D2.DMD mice showed reduced calcium green-5N fluorescence intensity and a significantly lower AUC, suggesting decreased mitochondrial calcium overload (Figure 1B,D).

**Figure 2:**
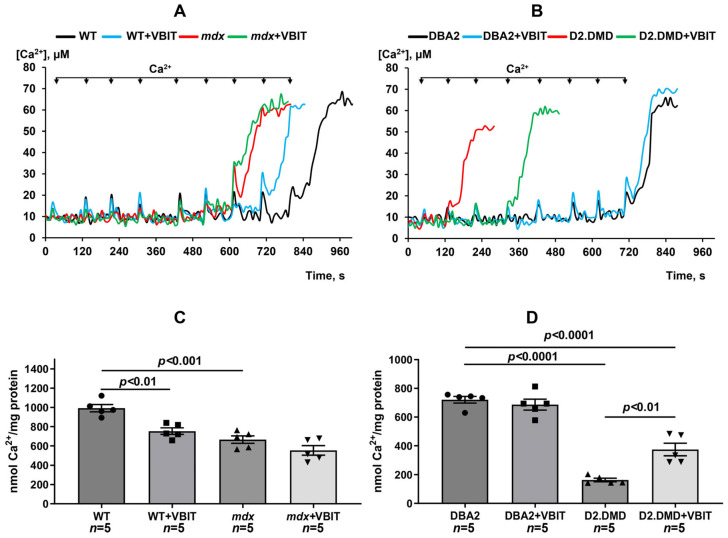
The impact of VBIT-4 on mitochondrial calcium. (**A**,**B**) Changes in the external [Ca^2+^] following the sequential addition of 20 μM Ca^2+^ pulses to suspensions of quadriceps mitochondria from the experimental animals. The data presented are from a representative experiment conducted on one mitochondrial sample. Similar results were obtained in four additional independent experiments. (**C**,**D**) Calcium retention capacity of the isolated quadriceps mitochondria. Data are expressed as mean ± SEM.

Mitochondrial calcium overload in the skeletal muscles of dystrophin-deficient mice was further associated with the reduced capacity of these organelles to absorb externally added calcium ions, indicating a decreased calcium retention capacity (Figure 2). This parameter also reflects the mitochondrial resistance to pathological induction of the calcium-dependent MPT pore. The reduction in the capacity to retain calcium was more pronounced in the novel D2.DMD mouse line compared with *mdx* mice. VBIT-4 treatment did not affect the calcium retention capacity in *mdx* mice. Interestingly, VBIT-4 significantly decreased this parameter in WT mice. Conversely, VBIT-4 increased the mitochondrial calcium retention capacity in D2.DMD mice (Figure 2). With the observed reduction in mitochondrial calcium overload, these results suggest improved calcium homeostasis in this severe DMD model.

### 2.2. VBIT-4 Reduces the Activity of Both the Mitochondrial and Tissue Fractions of Calpains, as Well as SR Stress Levels in the Quadriceps Muscles of D2.DMDel8-34 Mice but Not in Mdx Mice

**Figure 3:**
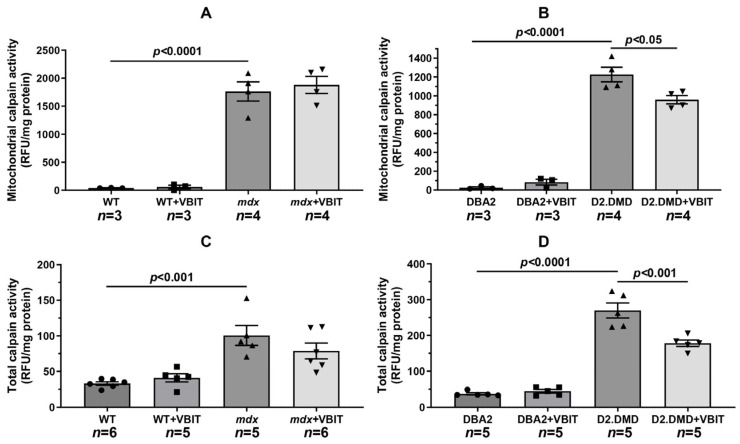
Calpain activity in quadriceps muscle mitochondria (**A**,**B**) and total quadriceps muscle extract (**C**,**D**) expressed as RFU/mg of protein. Data are expressed as mean ± SEM.

One of the key consequences of elevated calcium levels in dystrophin-deficient muscles is the excessive activation of Ca^2+^-dependent proteases (calpains), which contributes to the development of proteotoxic stress [34]. It should be noted that mitochondria also contain a calpain fraction whose activity depends on the calcium ion levels in the matrix. Overactivation of these mitochondrial calpains leads to impaired organellar protein function and mitochondrial dysfunction [35]. We assessed calpain activity in the mitochondrial and tissue extracts from mice. Our analysis revealed significantly increased mitochondrial calpain activity in both dystrophin-deficient mouse lines compared with their respective controls (Figure 3A,B), likely due to substantial calcium accumulation in the mitochondrial matrix of both *mdx* and D2.DMD mice. VBIT-4 treatment did not affect mitochondrial calpain activity in *mdx* mice but significantly reduced this parameter in D2.DMD mice. Furthermore, this VBIT-4 effect was accompanied by a marked decrease in the total calpain activity extracted from the quadriceps muscles of D2.DMD mice (Figure 3C,D).

**Figure 4:**
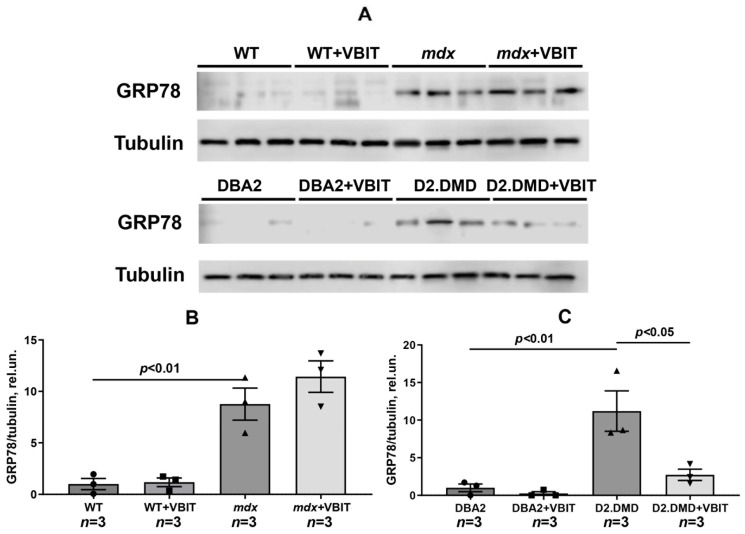
Western blotting of the GRP78 and tubulin (**A**), quantification of GRP78/tubulin ratio (**B**,**C**) in the skeletal muscles of the experimental groups of mice. Data are expressed as mean ± SEM.

Proteotoxic stress in dystrophin-deficient muscles triggers the activation of the SR stress response [36], as evidenced by the significantly elevated levels of the unfolded protein response (UPR) marker glucose-regulated protein 78 (GRP78) in the skeletal muscles of dystrophin-deficient mice (Figure 4). While VBIT-4 showed no significant effect on GRP78 levels in *mdx* mice, it demonstrated a pronounced beneficial effect in the more severe D2.DMD mouse model.

### 2.3. VBIT-4 Treatment Failed to Rescue Either the Deficient Oxidative Phosphorylation Capacity in the Quadriceps Mitochondria or the Elevated Oxidative Stress Observed in the Dystrophin-Deficient Muscle Tissue

Mitochondria from dystrophin-deficient skeletal muscles exhibit significantly reduced oxidative phosphorylation efficiency and ATP synthesis capacity [37,38]. This impairment is likely mediated by excessive Ca^2+^ accumulation in the mitochondrial matrix. Table 1 presents the respiratory parameters and oxidative phosphorylation capacity of the skeletal muscle mitochondria assessed by polarography. Mitochondria isolated from the quadriceps muscles of *mdx* mice showed significantly lower rates of ADP-stimulated respiration (State 3) and 2,4-dinitrophenol (DNP)-uncoupled respiration (State 3U_DNP_), which reflects the maximal electron transport chain activity. These changes were accompanied by a marked reduction in the respiratory control ratio (State 3/State 4), indicating impaired respiratory efficiency and oxidative phosphorylation compared with the wild-type controls. The suppression of oxidative phosphorylation was more pronounced in the novel D2.DMD mouse line, which displayed a severe pathological phenotype. In these animals, we observed substantially decreased oxygen consumption rates in both State 3 and State 3U_DNP_, along with reduced respiratory control ratios compared with *mdx* mice.

VBIT-4 treatment did not affect any measured parameters of mitochondrial respiration or oxidative phosphorylation in both dystrophin-deficient mouse lines. Interestingly, wild-type C57BL/10 mice treated by VBIT-4 showed decreased respiratory control ratios, suggesting potential impairment of oxidative phosphorylation efficiency in normal mitochondria.

**Figure 5:**
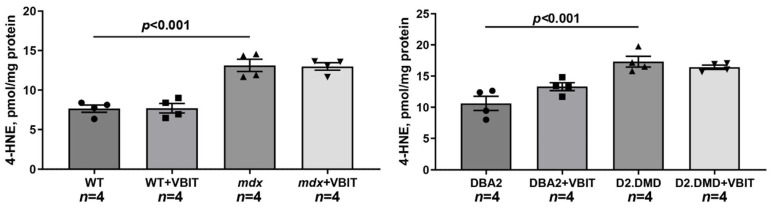
4-HNE levels in the quadriceps muscle extracts from the experimental mice. Data are expressed as mean ± SEM.

Dystrophin-deficient tissues generate substantial amounts of free radicals [39]. We assessed the levels of 4-hydroxy-trans-2-nonenal (4-HNE, a marker of oxidative stress) in skeletal muscle extracts and evaluated the effect of VBIT-4 on this parameter. As shown in Figure 5, 4-HNE levels were elevated in the muscles from both dystrophin-deficient mouse lines. However, VBIT-4 treatment did not affect 4-HNE levels in either *mdx* or D2.DMD mice.

### 2.4. VBIT-4 Improves the Mitochondrial Ultrastructure in D2.DMDel8-34 Mouse Quadriceps, with No Effect in Mdx Mice, but Impairs the Ultrastructure in Healthy Controls

**Figure 6:**
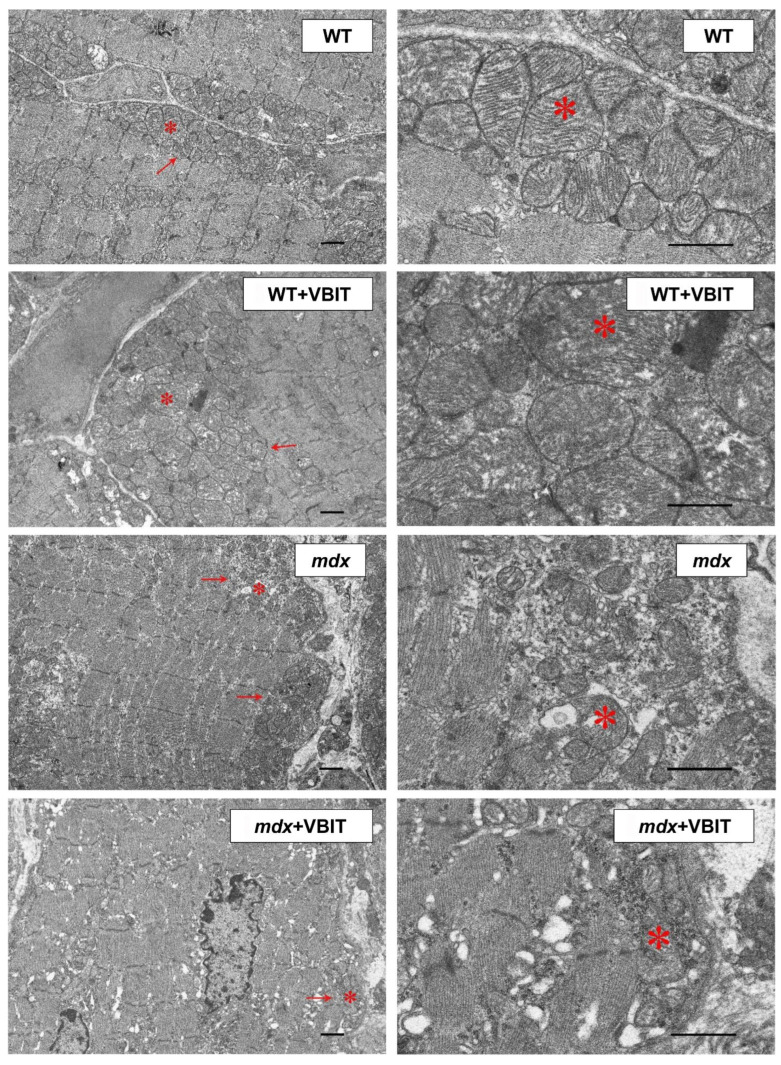
Representative transmission electron microscope images of the WT mouse quadriceps sections. Mitochondria within the subsarcolemmal population are indicated by red arrows. The asterisk highlights the same mitochondria at low (left side) and high (right side) magnifications. The scale bar represents 1 μm.

**Figure 7:**
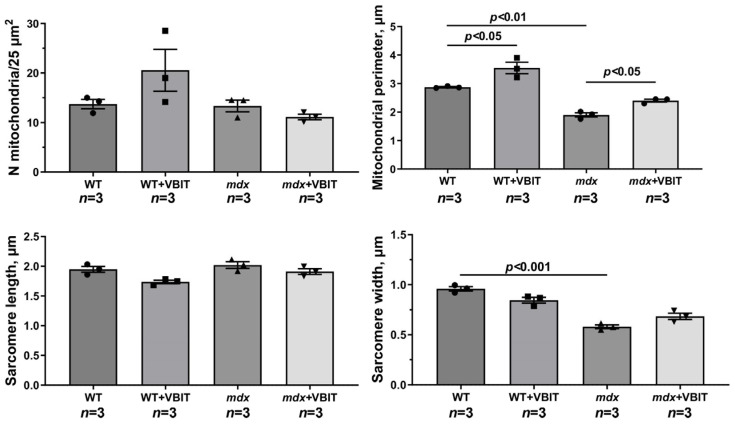
Electron micrograph (Figure 6) profiles: the number of mitochondria per plate, mitochondrial perimeter, sarcomere length, and sarcomere width. The number of fields of view examined in the groups ranges from 40 to 50. Data are expressed as mean ± SEM.

Skeletal muscle in dystrophin-deficient animals is characterized by a disrupted tissue ultrastructure, including alterations in the mitochondrial network. We investigated the effects of VBIT-4 on the quadriceps ultrastructure in mice. The study included an evaluation of sarcomere organization, mitochondrial apparatus integrity in the intermyofibrillar and subsarcolemmal regions, and SR morphology. In WT mice, the skeletal muscle exhibited a normal ultrastructure with well-organized sarcomeres and a typical mitochondrial distribution in the subsarcolemmal and intermyofibrillar zones (Figure 6). VBIT-4 administration induced mitochondrial alterations, namely, increased organelle number and size, swollen forms, while preserving the overall sarcomere structure (Figure 7). In *mdx* mice, electron microscopy revealed mosaic degenerative changes affecting myofibrils, sarcoplasm, SR, and mitochondria. The nuclei were frequently centralized, and the Z-lines were distorted, occasionally adopting a wavy or fragmented appearance. The myofibrils were thinner, with characteristic sarcomere narrowing (Figure 7). The intermyofibrillar spaces expanded due to the increased sarcoplasmic volume, containing elevated glycogen and lysosomes. The SR was markedly dilated, with proliferating and fragmenting cisternae forming vesicles around the mitochondria. The subsarcolemmal mitochondria were sparse, predominantly small and spherical, with significantly reduced perimeter compared to the controls (though their number remained unchanged). Some organelles displayed damage, disrupted cristae, matrix clearing, or outer membrane rupture.

In *mdx* + VBIT mice, the degenerative changes were less pronounced (Figure 6 and Figure 7). Centralized nuclei and sarcomere disorganization persisted, and the sarcomere width remained unaltered. However, a significant intermyofibrillar space expansion or sarcoplasmic volume increase was absent. SR proliferation was not observed, although focal cisternal swelling occurred. Mitochondrial number was unchanged, but severe damage, such as cristae destruction and localized matrix clearing was evident. It should be noted that the mean mitochondrial perimeter increased significantly in this group (Figure 7).

**Figure 8:**
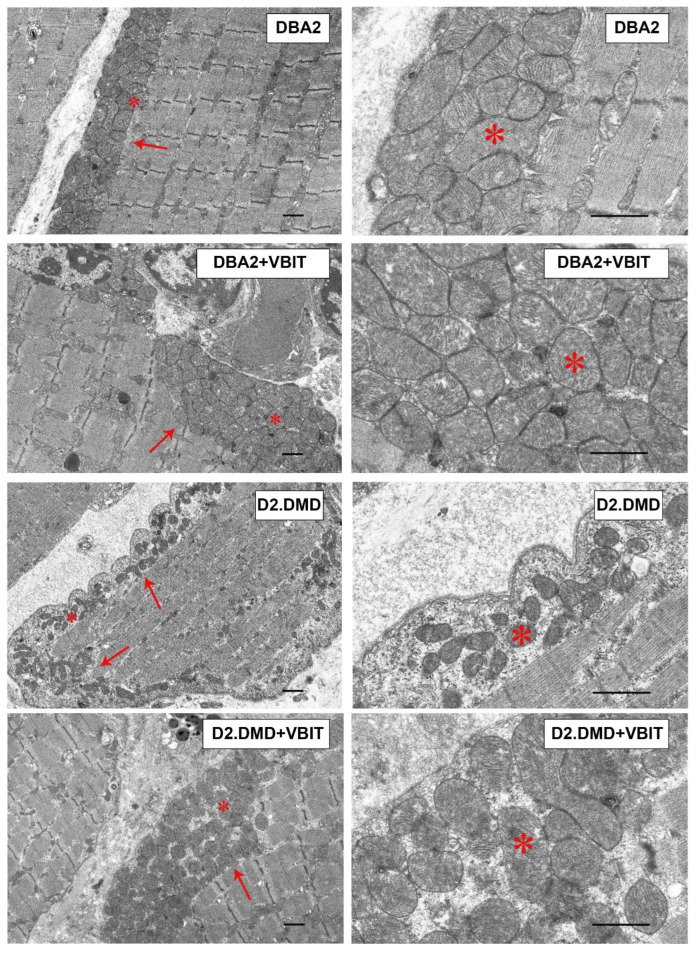
Representative transmission electron microscope images of the DBA/2 mouse quadriceps sections. Mitochondria within the subsarcolemmal population are indicated by red arrows. The asterisk highlights the same mitochondria at low (left side) and high (right side) magnifications. The scale bar represents 1 μm.

**Figure 9:**
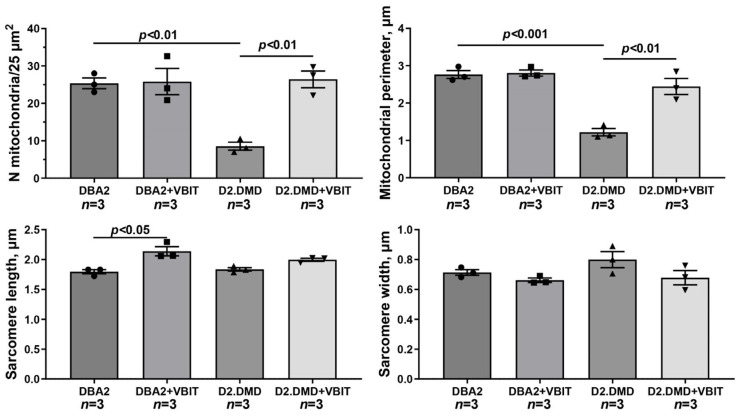
Electron micrograph (Figure 8) profiles: the number of mitochondria per plate, mitochondrial perimeter, sarcomere length, and sarcomere width. The number of fields of view examined in the groups ranges from 40 to 50. Data are expressed as mean ± SEM.

In the DBA/2 strain, moderate pathological changes were observed, including deformity of the Z-discs, abnormal mitochondria accumulation, and an increase in the extracellular matrix (Figure 8). VBIT-4 resulted in fragmentation of the SR in this group. The most pronounced degenerative changes were found in the D2.DMD mice. The nuclei were frequently positioned centrally, with some showing signs of fragmentation. Significant disruptions of the contractile apparatus were noted: thinning of myofibrils and disorganization of myofilaments. In severe cases, focal areas of complete degeneration with myolysis were present. The intermyofibrillar spaces were markedly expanded due to the increased sarcoplasmic volume, which showed elevated glycogen content and secondary lysosomes. Sarcomere length remained unchanged (Figure 9). The SR was sharply dilated. A characteristic feature was profound structural alterations of the sarcolemma, manifesting as numerous alternating invaginations and protrusions, within which deep invaginations of myocyte nuclei were occasionally found. The mitochondrial pool in the subsarcolemmal zone was significantly reduced (Figure 9), with the elongated forms exhibiting a rarefied matrix and reduced cristae predominating. The average organelle perimeter was significantly smaller compared with DBA group (Figure 9).

VBIT-4 did not alter the structure of the contractile elements or nuclei but substantially affected the mitochondria and SR in D2.DMD mouse quadriceps. In the subsarcolemmal zone, more mitochondria with a dense matrix and preserved cristae was observed, with their size matching controls (Figure 9). The SR lost its hyperplastic regions, acquiring a more flattened structure with predominant vesicles. Mitochondrial proliferation was noted in the intermyofibrillar spaces.

### 2.5. VBIT-4 Reduces Skeletal Muscle Calcification and Fibrosis in D2.DMD Mice and Exerts a Limited Beneficial Effect on Muscle Strength in These Animals

**Figure 10:**
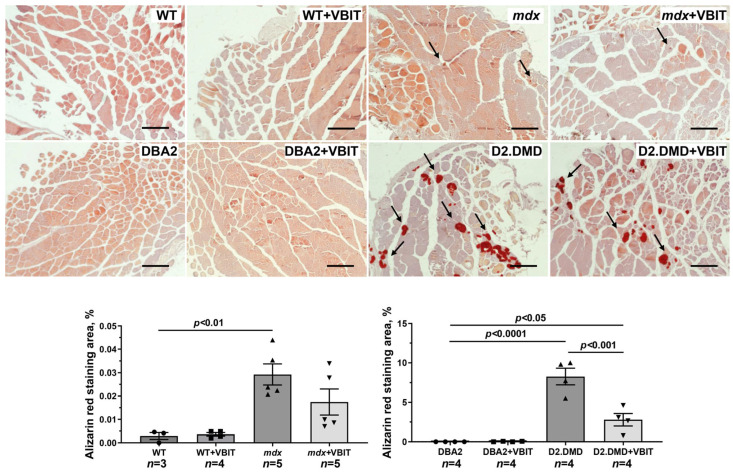
Representative histology images of the quadriceps muscles showing calcified regions (marked by arrows, Alizarin red staining) and the Alizarin red staining area. Scale bar is 50 μm.

**Figure 11:**
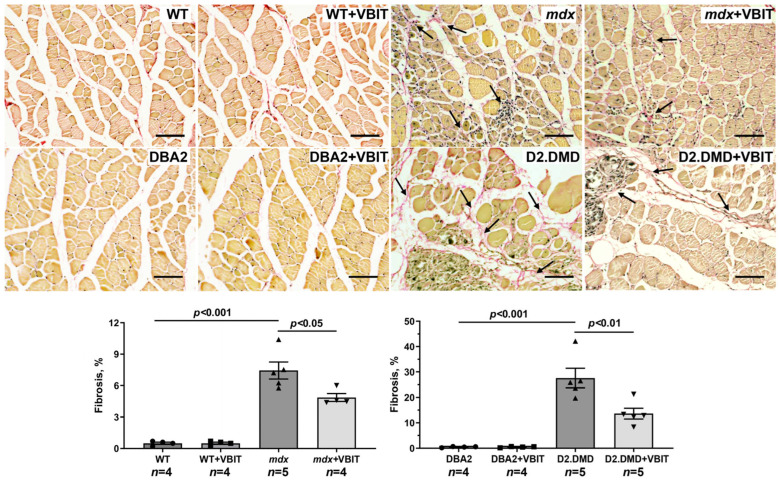
Representative histology images of the quadriceps muscles showing fibrotic regions (marked by arrows, Sirius red staining) and the Sirius red staining area. Scale bar is 50 μm.

**Figure 12:**
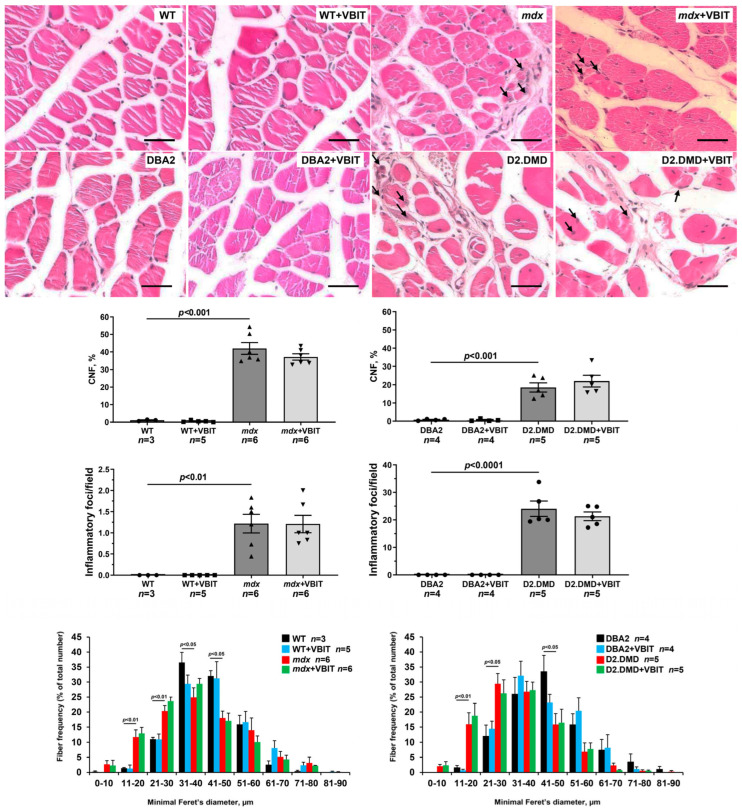
Representative H&E-stained sections showing centrally nucleated fibers (CNF) and examples of inflammatory infiltrates (indicated by arrows), the percentage of CNF, quantification of inflammatory foci per field and fiber size distribution (% of the total fiber number). Scale bar is 40 μm.

**Figure 13:**
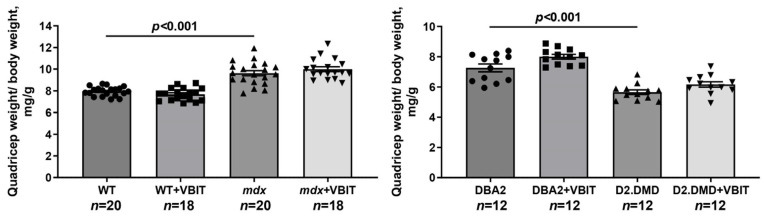
Relative mass of the quadriceps muscle in mice. Data are expressed as mean ± SEM.

**Figure 14:**
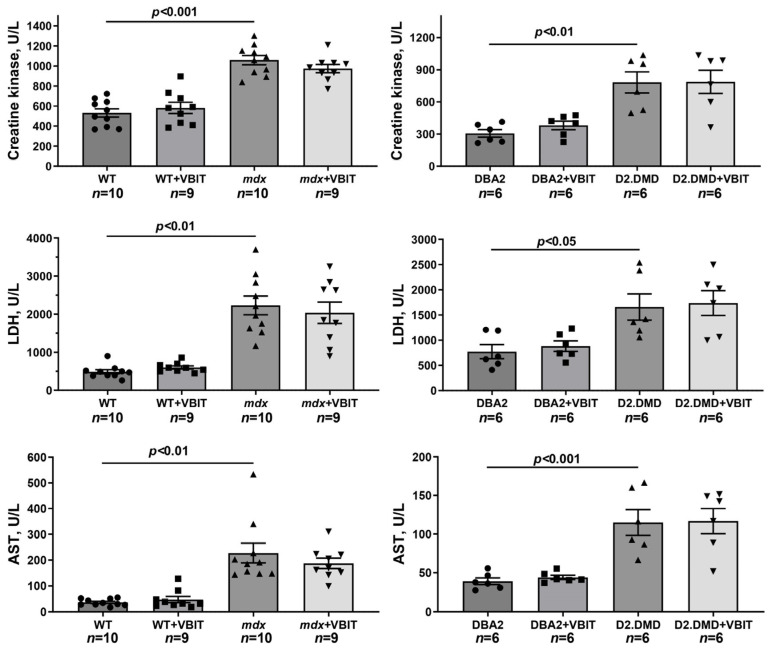
The levels of creatine kinase, LDH, and AST in the blood serum of experimental animals. Data are expressed as mean ± SEM.

In the final part of the study, we evaluated the effect of VBIT-4 on the key pathological manifestations of DMD. At the tissue level, one of the most prominent consequences of the disruption of calcium homeostasis in DMD is the ectopic calcification of skeletal muscles, caused by calcium phosphate deposition in the tissue [40]. Indeed, Alizarin red staining revealed significantly increased tissue calcification in the quadriceps of dystrophin-deficient mice, with this effect being hundreds of times more pronounced in the severe D2.DMD phenotype (Figure 10). The calcification of dystrophin-deficient muscle fibers promotes their degeneration and is accompanied by replacement with connective tissue, leading to fibrosis development that is also more severe in D2.DMD mice (Figure 11). Additionally, the quadriceps of dystrophin-deficient mice exhibited a significant increase in CNFs, characterized by nuclei positioned in the cytoplasmic center (Figure 12). CNFs represent regenerated myofibers, and their levels reflect the intensity of muscle fiber degeneration–regeneration cycles [41]. The CNF level was slightly lower in D2.DMD mice compared with mild *mdx* phenotype, likely reflecting the reduced regenerative potential in the former, presumably inherited from the DBA/2 mice. We also observed decreased mean minimal Feret’s diameter of quadriceps muscle fibers in *mdx* and D2.DMD mice compared with WT and DBA/2 controls, due to the increased proportion of small-diameter fibers (<30 μm) corresponding to regenerating fibers [42]. The high regenerative capacity of *mdx* mouse muscles was accompanied by pronounced quadriceps hypertrophy (Figure 13), while D2.DMD mice conversely showed reduced muscle mass (Figure 13). Among the most prominent diagnostic markers of DMD are elevated serum levels of skeletal muscle damage markers, including creatine kinase, lactate dehydrogenase (LDH), and aspartate aminotransferase (AST) assays. These enzymes were significantly increased in the dystrophin-deficient mice, reflecting significant damage to the muscle membrane and inflammatory activity (Figure 14). Also, to directly assess the inflammatory component of the pathology, we quantified inflammatory foci in the quadriceps muscle on H&E-stained sections. The number of inflammatory foci was significantly higher in both dystrophin-deficient models compared to their wild-type controls, with the severe D2.DMD mice exhibiting the most pronounced infiltration (Figure 12).

VBIT-4 treatment significantly reduced the Alizarin red-stained areas in the D2.DMD mouse muscles (Figure 10), accompanied by a marked decrease in fibrosis (Figure 11). In *mdx* mice, VBIT-4 showed only a weak tendency toward reduced calcification area (*p* = 0.2), although still with significant fibrosis reduction. However, VBIT-4 failed to affect the CNF levels, the number of inflammatory foci, fiber size distribution (Figure 12), or relative quadriceps mass (Figure 13). Furthermore, VBIT-4 treatment did not modify the elevated serum concentrations of muscle membrane damage markers in the dystrophin-deficient mice (Figure 14).

**Figure 15:**
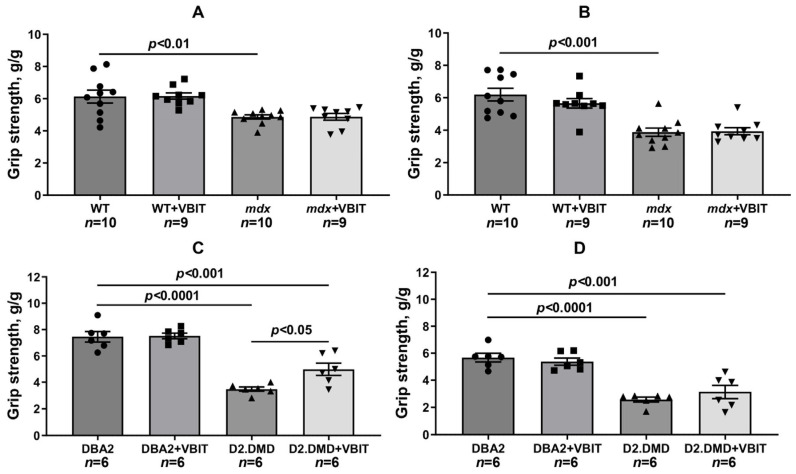
Grip strength in mice before (**A**,**C**) and after (**B**,**D**) 30 min treadmill running. Data are expressed as mean ± SEM.

**Figure 16:**
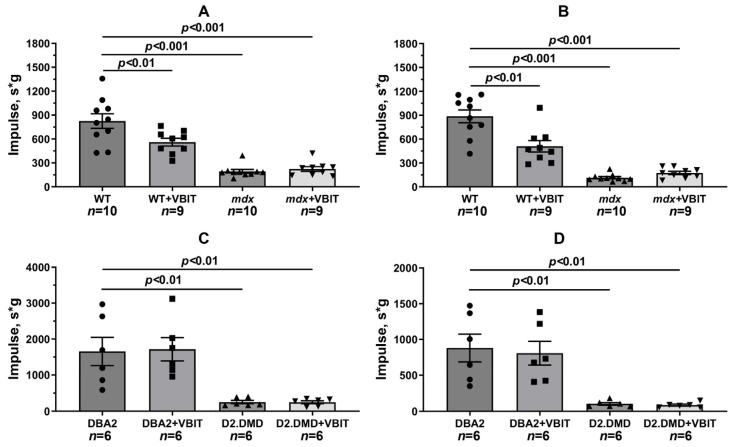
Holding impulse in mice before (**A**,**C**) and after (**B**,**D**) 30 min treadmill running. Data are expressed as mean ± SEM.

Functional tests revealed significant reductions in muscle strength (assessed by the grip strength test) and endurance (measured by the front paw wire holding impulse and post-exercise performance following 30 min treadmill running) in dystrophin-deficient animals (Figure 15 and Figure 16). To address the potential confounding effect of body weight on the holding impulse, we also analyzed the raw hanging time, a direct measure of fatigue resistance. The absolute hanging time data, presented in Supplementary Figure S1, were consistent with the holding impulse results. VBIT-4 showed no effect on grip strength or the holding time and impulse in *mdx* mice. Furthermore, VBIT-4 reduced the holding time and impulse in WT mice after exercise, suggesting the potential negative effects of this inhibitor on muscle function. However, VBIT-4 demonstrated a positive effect on muscle strength in D2.DMD mice: while these animals initially showed lower grip strength values, VBIT-4 administration led to a significant increase in this parameter. This effect was eliminated after the running session. One should note that VBIT-4 did not affect the holding time and impulse in D2.DMD mice. Thus, the impact of VBIT-4 on muscle strength and endurance in dystrophin-deficient animals appears to be limited.

## 3. Discussion

Mitochondrial dysfunction represents one of the secondary pathogenic mechanisms in DMD, contributing to the progressive degeneration of damaged muscle fibers. Our data demonstrate that mitochondria in dystrophin-deficient muscles exist in a state of chronic calcium overload (Figure 1). This condition increases organelle susceptibility to MPT pore opening (Figure 2), reduces oxidative phosphorylation efficiency (Table 1), and disrupts the mitochondrial ultrastructure (Figure 6, Figure 7, Figure 8 and Figure 9). It should be noted that mitochondrial dysfunction is significantly more pronounced in D2.DMD mice than in *mdx* mice, as evidenced by greater impairment in MPT pore resistance, oxidative phosphorylation efficiency, and morphological alterations, all of which are correlated with disease severity. For the first time, we have shown that mitochondrial dysfunction in dystrophin-deficient muscles may result from a catastrophic (>1000-fold) increase in mitochondrial calpain activity (Figure 3A,B). Recent evidence indicates that mitochondrial calpains (mitocalpains) play a crucial role in the selective removal of defective proteins and mitophagy, thereby maintaining organelle quality control [36]. However, under pathological calcium elevation in the matrix, mitocalpains can severely compromise both mitochondrial function and morphology through the proteolysis of mitochondrial proteins. Specific targets include complex I of the electron transport chain and ATP synthase, whose activity and protein levels are significantly reduced in dystrophin-deficient muscles [17,38]. Mitocalpains may also participate in MPT pore induction [35,43,44]. Of particular significance, our data reveal that mitocalpain activity in DMD exceeds the total tissue calpain activity in dystrophic muscles by approximately tenfold (Figure 3). This phenomenon may result from both locally high calcium concentrations and the potential absence of physiological calpain inhibitors (such as calpastatin) in the mitochondrial matrix [43]. These findings position mitocalpains, along with cytoplasmic calpains, as key contributors to degenerative processes in calcium-overloaded, dystrophin-deficient muscles.

Our results demonstrate that the modulation of mitochondrial function via VBIT-4, which inhibits the mitochondrial outer membrane voltage-dependent anion channel, improves mitochondrial calcium homeostasis in the dystrophin-deficient muscles of D2.DMD mice. Specifically, we observed reduced calcium overload (Figure 1) and increased resistance to MPT pore opening (Figure 2). This VBIT-4 effect may involve direct VDAC inhibition, as VDAC plays a crucial role in regulating calcium transport to the intermembrane space and participates in MPT pore formation. Its inhibition leads to the suppression of mitochondrial membrane potential generation (via blockade of substrate transport and electron transport chain inhibition), which is required for electrophoretic calcium uptake into the matrix [45]. In VBIT-4-treated D2.DMD mice, improved calcium homeostasis was accompanied by reduced mitocalpain activity (Figure 3A,B), which appears to contribute to better mitochondrial ultrastructure and normalization of the number and size of organelles in the muscle tissue (Figure 8 and Figure 9). However, these changes were insufficient to improve the oxidative phosphorylation efficiency (Table 1).

At the tissue level, VBIT-4 showed limited therapeutic effects. While it did not alter the abnormal sarcomere parameters (Figure 9) or elevated oxidative stress (as assessed by 4-HNE levels) (Figure 5) in dystrophin-deficient muscles, we observed some reduction in proteotoxic stress, evidenced by decreased total tissue calpain activity and SR stress marker GRP78 in D2.DMD mice (Figure 4A,C). The latter may indicate a reduced protein folding burden during VBIT-4 treatment. Moreover, we observed a decrease in the area of ectopic calcification in the skeletal muscles of the VBIT-4-treated D2.DMD mice (Figure 10). This effect of VBIT-4 may be attributed to a reduction in the number of calcium-overloaded mitochondria (Figure 1), which are known to act as foci of ectopic calcification and mineralization in soft tissues, including skeletal muscle [46,47]. A similar effect was recently observed upon the inhibition of mitochondrial calcium overload by MKT-077 in *mdx* mice [15]. The effect of VBIT-4 was accompanied by a decrease in fibrosis levels in the skeletal muscles of D2.DMD mice (Figure 11), a process known to frequently coincide with ectopic calcification of soft tissues [40]. At the same time, VBIT-4 had no effect on the intensity of muscle fiber degeneration, as assessed by the level of CNFs, inflammatory infiltration, muscle fiber size distribution (Figure 12), and relative muscle mass (Figure 13), nor did it affect fiber membrane integrity, as evaluated by the release of marker enzymes (creatine kinase, LDH, and AST) into the serum of mice (Figure 14).

It is important to note that the therapeutic effects of VBIT-4 were selective and observed exclusively in the severe D2.DMD model. This model specificity likely does not represent a contradiction but rather reflects the stage-dependent role of mitochondrial dysfunction in DMD progression. D2.DMD mice, with their severely impaired mitochondrial calcium retention capacity (Figure 2), suppressed oxidative phosphorylation (Table 1), and profound ultrastructural damage (Figure 8 and Figure 9), represent a condition where mitochondrial insufficiency becomes a substantial pathological driver. In this context, targeting VDAC and reducing mitochondrial calcium overload with VBIT-4 provides a clear beneficial effect, improving calcium homeostasis, reducing mitocalpain activity and ER stress, and subsequently mitigating calcification and fibrosis (Figure 1, Figure 2, Figure 3, Figure 4, Figure 10 and Figure 11). The preservation of muscle tissue contributed to a limited improvement in grip strength in these mice (Figure 15), although this effect was abolished after exercise, confirming the partial efficacy of the compound. In contrast, the lack of VBIT-4 effect in *mdx* mice can be explained by two key factors. First, mitochondrial dysfunction in *mdx* mice, although present, is less pronounced (Table 1, Figure 2) and may be insufficient to create a wide therapeutic window for VDAC inhibition. Second, and perhaps more importantly, the high regenerative capacity and metabolic adaptation in *mdx* muscles [48,49] may effectively compensate for the ongoing low-grade mitochondrial impairment, masking the potential benefit of VBIT-4. Furthermore, it should be noted that we observed no reduction in serum creatine kinase levels (Figure 14), inflammatory infiltration (Figure 12), oxidative stress (Figure 5), or the proportion of regenerating fibers (Figure 12). This pattern clearly demonstrates that VBIT-4 targets a specific consequence of dystrophin deficiency, namely, mitochondrial calcium overload, without affecting other parallel pathways (such as sarcolemmal fragility, chronic inflammation, and elevated ROS production). This is likely because under pathological conditions, VBIT-4 primarily improves calcium homeostasis without addressing other DMD pathogenic factors. The lack of effect on oxidative phosphorylation also suggests that mitochondrial dysfunction in DMD has a multifactorial nature requiring a combined therapeutic approach.

We also observed that VBIT-4 administration induced adverse effects on mitochondrial function and ultrastructure in healthy WT mice. Specifically, VBIT-4 reduced the oxidative phosphorylation efficiency (Table 1) and calcium retention capacity (Figure 2A,C) in quadriceps mitochondria, and caused ultrastructural alterations, including mitochondrial swelling (Figure 7). This phenomenon is not entirely unexpected. Given that VBIT-4 is an inhibitor of VDAC, a key channel regulating the flux of metabolites (including ATP/ADP) and ions across the outer mitochondrial membrane, its chronic inhibition in healthy tissues could disrupt normal mitochondrial energetics and calcium buffering capacity [45,50]. In a healthy physiological context, where mitochondrial calcium overload is absent, such disruption of VDAC function likely outweighs any potential benefit, leading to a suboptimal energy state and the observed functional impairments, including reduced endurance (Figure 16A,B and Figure S1A,B). This contrast underscores the therapeutic window of VBIT-4, whose benefits manifest specifically in pathological conditions of severe calcium overload (as in D2.DMD mice), while in normocalcemic conditions, it may perturb baseline mitochondrial function.

Our data confirm that VDAC targeting may represent a promising therapeutic direction for severe DMD forms, as recently demonstrated for another VDAC inhibitor olesoxime in a similar severe D2-*mdx* model [25]. Moreover, these and previous findings not only confirm the efficacy of VDAC modulation but also suggest that generally improving mitochondrial calcium homeostasis and reducing organelle calcium overload could enhance the structure and function of dystrophin-deficient muscles. Broadly, similar effects, with varying efficacy, might be achieved by modulating calcium transport at SR/mitochondria contact sites, particularly through the regulation of SR inositol trisphosphate receptor (IP_3_R) [13,14], and suppression of GRP75 protein activity [15] that mediates IP_3_R-VDAC linkage. However, this approach likely requires a combination with other drugs targeting oxidative stress reduction, muscle regeneration enhancement, and other manifestations of this complex pathology. Additionally, further studies are needed, including the following: investigation of VBIT-4 long-term effects, testing different drug doses and administration regimens, and evaluation of combinations with existing therapeutic strategies (like glucocorticoids).

## 4. Materials and Methods

### 4.1. Animals

The male C57BL10 mice (wild type, WT), dystrophin-deficient *mdx* mice (C57BL/10ScSn-*mdx*), and DBA/2 mice were obtained from the Animal Breeding Facility, Branch of the Shemyakin and Ovchinnikov Institute of Bioorganic Chemistry, Russian Academy of Sciences, Russia (IBCh RAS Unique Research Device “Bio-model”, Pushchino, Russia). Dystrophin-deficient mice of the D2.DMDdel8-34 line were obtained at the Laboratory of Modeling and therapy of hereditary diseases, Institute of Gene Biology RAS. For this purpose, homozygous females of DMDdel8-34 [33] were crossed with males of the DBA/2 line, which are characterized by reduced efficiency of muscle fiber regeneration. The resulting generation was taken as F0. Four backcrosses with mice of the DBA/2 line were then performed for the obtained offspring. For the current study, F4 males were used. Male mice of the DBA/2 genotype from the same litters were used as the control group. All mice were maintained under a controlled 12 h photoperiod, and water and food were provided ad libitum.

N-(4-Chlorophenyl)-4-hydroxy-3-(4-(4-(trifluoromethoxy)phenyl)piperazin-1-yl)butanamide (or VBIT-4) was obtained from BLD Pharmatech Ltd. (Shanghai, China). VBIT-4 was dissolved in a 5:5:10:80 (v/v) mixture of Cremophor/DMSO/ethanol/PBS and administered intraperitoneally at a dose of 20 mg/kg body weight, with injections given every other day for 4 weeks, resulting in a total of 14 injections. Similar doses of VBIT-4 had been previously used in models of Alzheimer’s disease [27] and systemic lupus erythematosus [28].

**Figure 17:**
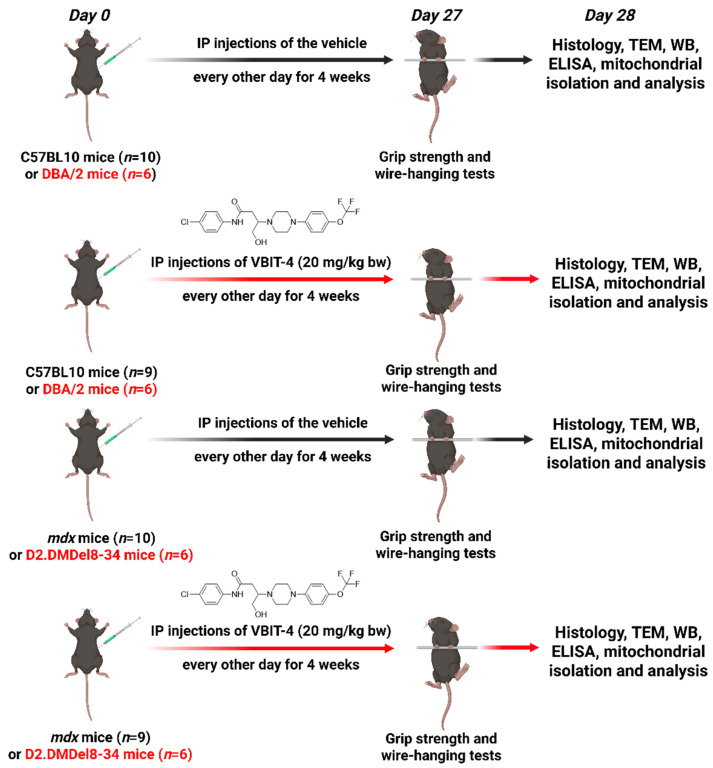
The experimental design of the study.

For this study, we used 4-week-old *mdx* mice and wild-type C57BL/10 mice (control group), which were randomly divided into 4 experimental groups: wild-type (WT) (*n* = 10), wild-type treated by 20 mg/kg VBIT-4 (WT + VBIT) (*n* = 9), *mdx* (*n* = 10), and *mdx* mice treated by 20 mg/kg VBIT-4 (*mdx* + VBIT) (*n* = 9). In a parallel study, we examined 4-week-old D2.DMDel8-34 mice and DBA/2 control mice, which were similarly randomized into 4 groups: control (DBA2) (*n* = 6), control treated by 20 mg/kg VBIT-4 (DBA2 + VBIT) (*n* = 6), D2.DMDel8-34 mice (D2.DMD) (*n* = 6), and D2.DMD mice treated by 20 mg/kg VBIT-4 (D2.DMD + VBIT) (n = 6). Control animals were treated by intraperitoneal injections of the vehicle solution alone. A brief research protocol is shown in Figure 17.

At the end of the treatment period, the mice were euthanized by cervical dislocation under anesthesia induced by a zoletil/xylazine mixture. Blood samples were collected in 1.5 mL Eppendorf tubes and allowed to coagulate at room temperature before centrifugation at 800× *g* for 10 min at 4 °C. The resulting serum was aliquoted and stored at −80 °C for subsequent biochemical analyses. Fresh quadriceps muscle samples (*vastus lateralis*) were immediately harvested for histological and electron microscopic examination. Mitochondrial isolation procedures were performed using fresh skeletal muscle tissue samples comprising the entire quadriceps from both hindlimbs.

### 4.2. Grip Strength and Wire-Hanging Tests

The muscle strength of the animals was evaluated using the grip strength test (IITC Life Science, Woodland Hills, CA, USA). The mice were familiarized with the test one week before sacrifice. Testing was conducted the day before the animal’s sacrifice. The results were expressed as grams per animal body weight. The mean result from each of the three trials per mouse was utilized for the analysis.

The endurance of the mice was evaluated through a wire-hanging test. The mice were familiarized with the test one week before being sacrificed. The final testing was conducted the day before the sacrifice, occurring 1 h after the grip strength test and following the same sequence of animals. The testing was conducted on a setup comprising a 3 mm thread string, 38 cm in length, and 49 cm above a padded surface. The maximum hanging time (s) was recorded as a direct measure of endurance. The holding impulse [(s × g) = hanging time (s) × body weight (g)] was used to correct for the negative effects of body weight on the hanging time and represented the minimal sustained tension (impulse) developed by a mouse to support itself against gravity for a specified time [51].

To properly assess endurance, immediately after the initial tests, the mice underwent a 30 min running session on a treadmill (Vetbot, Dorogobuzh, Russia) at a speed of 12 m/min, in accordance with established protocols [52]. Following this exercise, a second round of animal testing was conducted using the same sequence of evaluations.

### 4.3. Creatine Kinase, LDH, and AST Assays

The activities of creatine kinase, AST, and LDH in mouse blood serum were analyzed spectrophotometrically at 340 nm using a Multiscan Go plate spectrometer (Thermo Fisher Scientific, Waltham, MA, USA) and a commercially available test kit (Vector-Best, Novosibirsk, Russia).

### 4.4. Transmission Electron Microscopy

Tissue samples from the mouse quadriceps (*vastus lateralis*, three samples per group from random animals, whose tissue was also collected for histology) were processed as detailed in a previously published study [11]. Semi-thin sections were initially prepared to identify the mid-belly region and the muscle’s transverse orientation, as in [53], using the EVOS M5000 microscope (Thermo Fisher Scientific, Waltham, MA, USA). Sections of 60–70 nm thickness were cut from solid epon blocks using a Leica EM UC6 ultramicrotome (Leica, Wetzlar, Germany). The sections were then stained with uranyl acetate and lead citrate before being imaged using a JEM-1400 electron microscope (Leica, Wetzlar, Germany). The images were analyzed using the ImageJ software, version 1.53c (National Institutes of Health, Bethesda, MD, USA). Mitochondrial morphometry was achieved through manual tracing of cross-sections along the outer membrane of the organelles. A total of 150 cross-sectional profiles of mitochondria, collected at 50 per sample, were obtained for each group of mice. The average from each mouse was included in the analysis.

### 4.5. Histological Analysis

The *vastus lateralis* of the mice was embedded in paraffin following pre-treatment as detailed earlier [11]. The blocks were then sectioned into 5 μm serial sections using a Minux S710 rotary microtome (RWD, Shenzhen, China). The obtained slides were stained with hematoxylin and eosin (H&E) to gauge the severity of histological alterations and with Sirius red (GC307014, Servicebio, Wuhan, China) to evaluate the extent of fibrosis. Alizarin red S (Interchim, Russia) was used for detecting the calcium deposits. The images (muscle mid-belly region identified as described previously [53]) were examined using the EVOS M5000 microscope (Thermo Fisher Scientific, Waltham, MA, USA). Images were captured in a transmitted light channel and further examined with ImageJ software, version 1.53 (National Institutes of Health). Inflammatory foci per field were scored in five high-power (40×) non-overlapping fields in a blinded manner. The minimal Feret’s diameter was measured using the XP-Pen graphic tablet (XP-PEN, Shenzhen, China) and the ImageJ software’s freehand selection tool in the same fields. The area of fibrosis in the connective tissue was quantified using an XP-Pen graphic tablet and the freehand selection tool of Image J software, and expressed as a percentage of the tissue area stained with Sirius red in relation to the total section area. The area of the calcified tissue was measured using the XP-Pen graphic tablet and the freehand selection tool of the Image J software, and then it was expressed as a percentage of the stained tissue area relative to the total section area. The average from each mouse was included in the analysis, with at least 10 serial sections per animal considered.

### 4.6. Electrophoresis and Western Blotting

A total protein extract was prepared using 10 mg of frozen quadriceps tissue. For integrity and functionality, a complete protease inhibitor cocktail (P8340, Sigma-Aldrich, USA), a phosphatase inhibitor cocktail (P0044 Sigma-Aldrich, St. Louis, MO, USA), Na_3_VO_4_ (1 mM), PMSF (1 mM), EDTA (1 mM), and EGTA (1 mM) were used. Protein extraction was performed using RIPA buffer (20–188, Merck Millipore Ltd., Billerica, MA, USA). The Bradford assay (Bio-Rad Laboratories, Hercules, CA, USA) was utilized to quantify the protein amount. The samples were diluted in Laemmli buffer, subjected to 12.5% SDS–PAGE (10 µg/lane), and then transferred to a 0.45 µm nitrocellulose membrane (Cytiva, Marlborough, MA, USA). The membrane was then incubated with the required primary antibody, Anti-GRP78 antibody (AF5366), which was sourced from Affinity Biosciences (Cincinnati, OH, USA). The anti-tubulin antibody (AC015) was sourced from Abclonal (Wuhan, China). Secondary horseradish peroxidase-conjugated antibodies (7074), obtained from Cell Signaling Technology Inc. (Danvers, MA, USA), were used to detect immunoreactivity. ECL reagents from Pierce (Rockford, IL, USA) were utilized to assess peroxidase activity. Proteins were visualized and measured using the LI-COR system (LI-COR, Lincoln, NE, USA) and the LI-COR Image Studio software (version 5.2.5), and adjusted to tubulin, which was used as a loading control reference.

### 4.7. Measurement of 4-HNE in the Quadriceps Tissue

4-HNE levels in the mouse quadriceps were measured by ELISA using a commercially available test kit (Elabscience, Wuhan, China) and a Multiskan Go plate reader (Thermo Fisher Scientific, Waltham, MA, USA).

### 4.8. Isolation of Skeletal Muscle Mitochondria and Their Functional Analysis

The differential centrifugation method was used to isolate mitochondria from the total quadriceps of both the hind limbs of mice [54]. The Bradford assay revealed 20–30 mg of mitochondrial protein per mL in the resulting suspension. The rate at which mitochondria consume O_2_ was measured using an Oxygraph Plus system (Hansatech Instruments, King’s Lynn, UK), and the incubation buffer contained 120 mM KCl, 5 mM NaH_2_PO_4_, 10 mM HEPES-KOH (pH 7.4), and 2.5 mM potassium malate and 2.5 mM potassium glutamate. Each assay used about 0.3 mg of mitochondrial protein/mL, 200 μM ADP, and 50 μM 2,4-dinitrophenol (DNP). The rate of oxygen consumption was expressed in units of nmol O_2_/min/mg mitochondrial protein, and the respiratory control ratio (RCR) was defined as the ratio of the oxygen consumption rate during state 3 to that during state 4 [55].

A suspension of 2 mg of mitochondrial protein per mL was prepared in a solution consisting of 210 mM mannitol, 70 mM sucrose, 1 mM KH_2_PO_4_, 10 μM EGTA, and 10 mM HEPES-KOH buffer at pH 7.4, and was supplemented with 250 nM calcium green-5N (Invitrogen, Carlsbad, CA, USA). A concentration of 40 µM alamethicin (ALM) was utilized to induce non-specific permeabilization of the mitochondrial membranes and calcium release, which was accompanied by a sharp increase in calcium green-5N fluorescence [10].

The mitochondrial Ca^2+^ retention capacity (CRC) was evaluated using the calcium-sensitive indicator arsenazo III, with absorbance measurements taken at 675 and 685 nm on a Multiskan Go spectrometer (Thermo Fisher Scientific) according to the previously published methodology [12]. The assessment was conducted using the same experimental system and incubation medium, but with 50 μM arsenazo III substituted for calcium green-5N and a mitochondrial protein concentration of approximately 0.2 mg/mL. For the measurement, calcium chloride was administered in pulses of 20 μM until the spontaneous release of calcium ions from the mitochondrial matrix was detected, indicating the opening of the MPT pore in the inner mitochondrial membrane.

### 4.9. Measurement of Calpain Activity in the Tissue and Mitochondria

Activated calpain levels in the protein extracts (0.1 mg protein/mL) were quantified using a commercial calpain activity assay kit (#ab65308, Abcam, Cambridge, MA, USA). Quadriceps muscle tissue or isolated quadriceps mitochondria were homogenized in an extraction buffer specifically formulated to prevent calpain auto-activation during sample preparation. The calpain enzymatic activity was determined fluorometrically using the synthetic substrate Ac-LLY-AFC. Following 1 h of incubation according to the manufacturer’s protocol, the activity was expressed as relative fluorescence units (RFU) per mg of protein.

### 4.10. Statistical Analysis

The results are shown as mean ± SEM. Data analysis was performed using GraphPad Prism 10, specifically through one-way ANOVA and Tukey’s multiple comparison post-test after the analysis. A *p*-value threshold of 0.05 was set to determine statistical significance. The sample size *n* for all data represents the number of biologically independent animals per group, with the exception of the relative quadriceps mass data (Figure 13), for which *n* denotes the number of hindlimbs analyzed.

## 5. Conclusions

**Figure 18:**
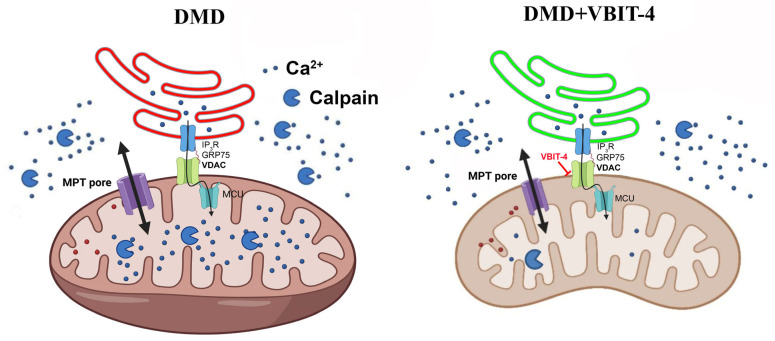
Schematic summary of the therapeutic effects of VBIT-4 in a severe model of Duchenne muscular dystrophy. VBIT-4 inhibition of VDAC improves mitochondrial calcium (Ca^2+^) turnover, reduces the activity of mitochondrial calpains and ER stress, leading to decreased muscle calcification and fibrosis, and results in partial improvement of muscle function.

The present study demonstrates that the VDAC inhibitor VBIT-4 can improve the mitochondrial state and ultrastructure while slowing the pathology progression in mice with a severe DMD phenotype (Figure 18) but shows no significant effect in mild *mdx* model. These results highlight the crucial role of mitochondrial calcium imbalance in DMD progression and open new possibilities for developing targeted therapeutic strategies—particularly relevant given the current absence of effective genetic therapies.

## Figures and Tables

**Table 1:** Impact of VBIT-4 on respiration and oxidative phosphorylation in mouse skeletal muscle mitochondria.

Group	Respiratory Rate, nmol O_2_/min per 1 mg of Protein				RCR (Relative Units)
	State 2	State 3	State 4	State 3U_DNP_	
WT (*n* = 5)	33.2 ± 3.5	194.5 ± 5.4	30.3 ± 1.6	256.8 ± 11.8	6.5 ± 0.3
WT + VBIT (*n* = 5)	28.9 ± 2.4	146.0 ± 18.9	34.0 ± 2.3	188.3 ± 24	4.3 ± 0.4 **
*mdx* (*n* = 5)	31.2 ± 1.9	136.9 ± 10.8 *	28.9 ± 1.9	173.4 ± 14.1 *	4.7 ± 0.2 **
*mdx +* VBIT (*n* = 5)	31.6 ± 1.8	154.3 ± 11.8	35.5 ± 2.3	191.8 ± 19.5	4.4 ± 0.3 **
DBA2 (*n* = 4)	27.2 ± 0.8	145.5 ± 5.5	29.4 ± 1.8	176.3 ± 3.4	5.0 ± 0.2
DBA2 + VBIT (*n* = 4)	24.7 ± 1.9	136.4 ± 5.5	31.2 ± 3.3	161.4 ± 9.5	4.5 ± 0.4
D2.DMD (*n* = 4)	29.9 ± 1.4	61.0 ± 10.9 **	40.6 ± 3.4	83.0 ± 11.6 **	1.6 ± 0.4 **
D2.DMD + VBIT (*n* = 4)	27.4 ± 1.6	67.3 ± 9.8 **	31.6 ± 2.8	92.1 ± 11.8 **	2.1 ± 0.2 **

The assay buffer consisted of 130 mM KCl, 5 mM NaH_2_PO_4_, 10 μM EGTA, and 10 mM HEPES-KOH (pH 7.4). Glutamate at a concentration of 2.5 mM and malate at 2.5 mM were used as respiratory substrates. State 3 mitochondrial respiration was initiated by the addition of 200 μM ADP; state 3U_DNP_ (uncoupled) respiration was induced by 50 μM DNP. Data are expressed as mean ± SEM. Statistical significance was assessed as * *p* < 0.05 versus the control group (WT or DBA2) and ** *p* < 0.01 versus the control group (WT or DBA2).

## Data Availability

The data presented in this study are available upon request from the corresponding author.

## Supplementary Materials

The following supporting information can be downloaded at: https://www.mdpi.com/article/10.3390/ijms26188845/s1.

## Abbreviations

The following abbreviations are used in this manuscript:

DMD	Duchenne muscular dystrophy
VDAC	Voltage-dependent anion channel
MPT	Mitochondrial permeability transition
SR	Sarcoplasmic reticulum
CRC	Calcium retention capacity
4-HNE	4-hydroxy-trans-2-nonenal
GRP78	Glucose-regulated protein 78
RCR	Respiratory control ratio
DNP	2,4-dinitrophenol
CNF	Centrally nucleated fibers
AST	Aspartate aminotransferase
LDH	Lactate dehydrogenase
UPR	Unfolded protein response
ROS	Reactive oxygen species
