# Supplementary material for: VBIT-4 Rescues Mitochondrial Dysfunction and Reduces Skeletal Muscle Degeneration in a Severe Model of Duchenne Muscular Dystrophy
Source: Int J Mol Sci. 2025 Sep 11;26(18):8845. doi: 10.3390/ijms26188845 (PMC12469774; doi:10.3390/ijms26188845)
Supplement: Supplementary file 1 [file ijms-26-08845-s001.zip › ijms-3854173-supplementary.pdf]

International Journal of
Molecular Sciences

Article

VBIT-4 Rescues Mitochondrial Dysfunction and Reduces Skeletal Muscle Degeneration in a Severe Model of Duchenne Muscular Dystrophy

Mikhail V. Dubinin [1,*], Anastasia E. Stepanova [1], Irina B. Mikheeva [2], Anastasia D. Igoshkina [1], Ekaterina N. Kraeva [1], Alena A. Cherepanova [1], Eugeny Yu. Talanov [3], Anna V. Polikarpova [4,5], Maxim E. Astashev [6,7], Vyacheslav A. Loginov [4,5] and Tatiana V. Egorova [4,5]

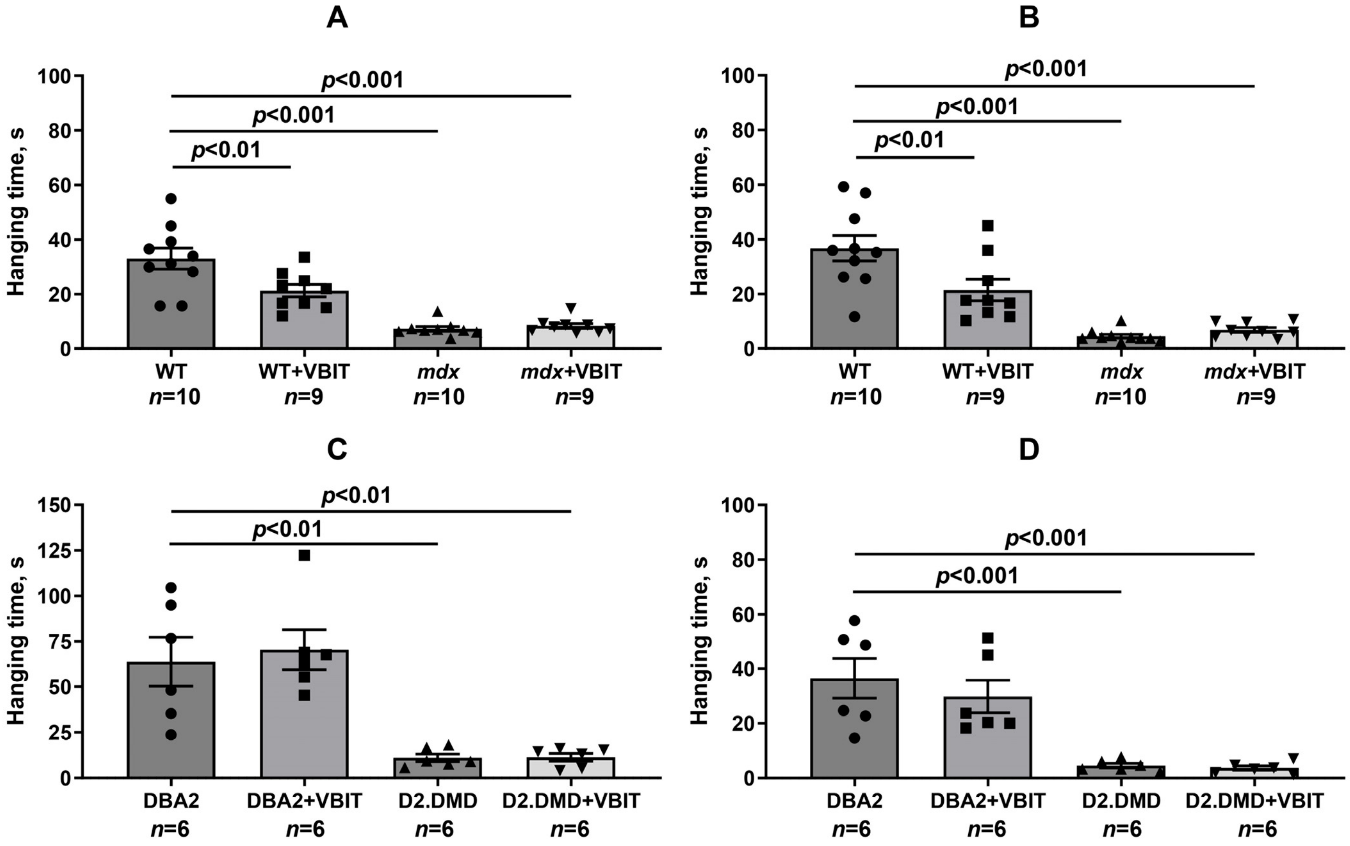

Figure S1. Wire hanging time of mice before (A), (C) and after (B), (D) a 30 min treadmill run. Data are expressed as mean ± SEM.
